# An Interesting Case of Takayasu Arteritis With Acute Bilateral Pulmonary Thromboembolism

**DOI:** 10.7759/cureus.47944

**Published:** 2023-10-30

**Authors:** Jolsana Augustine, Mohammed Harriss, Yogeeswari Satyanarayanan

**Affiliations:** 1 Pulmonology, Medcare Hospital Sharjah, Sharjah, ARE; 2 Cardiology, Medcare Hospital Sharjah, Sharjah, ARE

**Keywords:** thromboembolism, computed tomographic angiography, pulseless disease, large vessel vasculitis, acute pulmonary embolism, takayasu arteritits

## Abstract

Takayasu arteritis (TA) is an autoimmune vasculitis with unknown etiology. It can have varied presentations ranging from nonspecific symptoms to florid vasculitic symptoms. Awareness of the complications of this disease is also vital in managing patients who are already diagnosed with TA. We present the interesting case of a middle-aged woman, diagnosed case of TA who presented with an acute pulmonary embolism masquerading as an acute lower respiratory infection. Delayed diagnosis or misdiagnosis of acute major thromboembolism can be fatal. There needs to be a high index of suspicion from the clinician's end to reach a diagnosis and prompt intervention.

## Introduction

Takayasu arteritis (TA) is a large vessel vasculitis characterized by granulomatous inflammation of the vessel wall. The etiopathogenesis of this disease is still unclear. TA predominantly affects young females during the second or third decades of life [1]. It mainly involves large arteries like the aortic arch and its primary branches. Patients usually experience claudication and extremity pain and on clinical examination may reveal bruits, absent or diminished pulses, and loss of blood pressure. These are due to segmental occlusion by the vasculitic process and that is why TA is labeled as a pulseless disease. It has an insidious course generally. However, the disease can have dramatic presentations like acute loss of vision or stroke.

## Case presentation

A 45-year-old woman from Pakistan presented with worsening breathing difficulty, new onset cough, and fever for three days. She had some breathlessness for the past one year while hurrying or climbing stairs; modified Medical Research Council (mMRC) grade 1 worsened over the last month to mMRC grade 3. For the last two days, she was breathless even at rest. Her dyspnea and cough were not associated with any nocturnal or seasonal variation or wheezing. She experienced chest tightness along with dyspnea with no history of any radiating pain elsewhere. No relation with food intake was noted. She had worsening dyspnea on lying down for the past two days. She gives no history of substance abuse. She is a homemaker.

She was diagnosed with TA at the age of 19 years in Pakistan and was on mycophenolate and steroids until 2017 after which she was labeled as clinically stable Takayasu disease in remission. Her last magnetic resonance angiography report revealed bilateral carotid artery, right vertebral artery, and right subclavian artery stenosis (images not available). She could perform all routine household chores at ease before this current presentation. Two weeks back she presented to a hospital in Karachi. She was prescribed inhaled bronchodilators but she did not experience any betterment. With this background, she presented to our pulmonary medicine outpatient department in Medcare Hospital, Sharjah.

She was febrile with a temperature of 38°C and tachypneic (respiratory rate of 34/min) at presentation. Her Sp0_2_ level was 91% on room air. A general examination showed bilateral pitting pedal edema. No neck venous engorgement was noted. Auscultation revealed bilateral crackles. No murmurs were heard over the carotid, aortic, or pulmonary areas.

**Figure 1:**
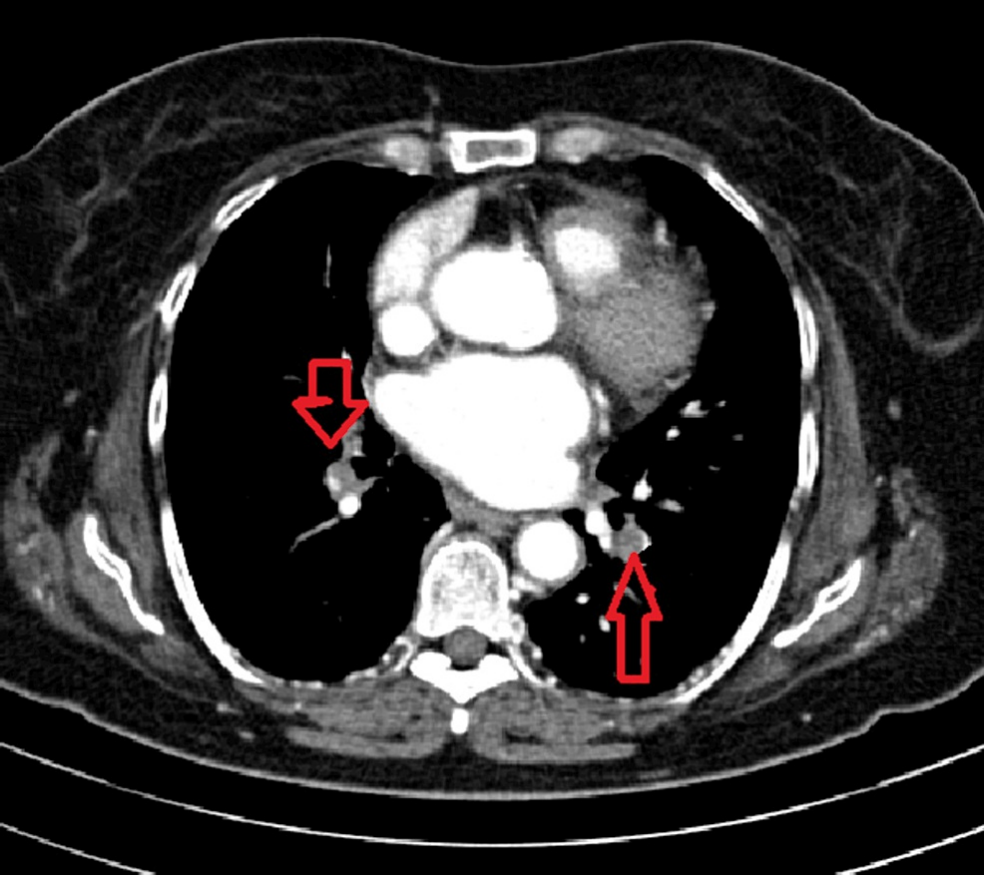
Cross-sectional image showing pulmonary embolism bilaterally Arrows clearly demonstrate filling defects in pulmonary vasculature

**Figure 2:**
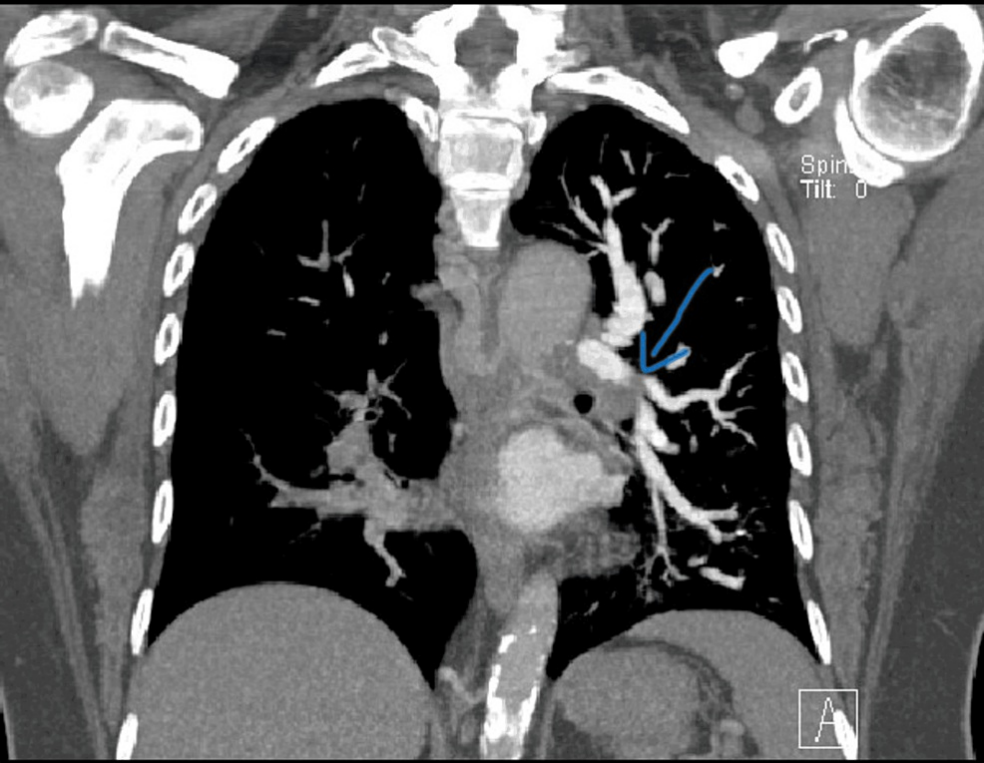
Sagittal image showing pulmonary embolism Arrow highlighting the absence of contrast showing embolism in pulmonary vasculature

The results of the investigations showed that the complete blood count, renal function tests, procalcitonin, and electrolytes were all within normal range. D-dimer was elevated at 1.58 ug/mL (normal range: 0.00-0.50ug/mL). The patient's liver function tests indicate elevated levels with alanine transaminase (ALT) at 55 U/L (normal range: 0.00-33.00 U/L), aspartate aminotransferase (AST) at 60 U/L (normal range: 0.00-33.00 U/L), erythrocyte sedimentation rate (ESR) at 45 mm/hr (normal range: 0-12 mm/hr), C reactive protein at 180 mg/l (normal range: 0.00-5 mg/l), and B-type natriuretic peptide (BNP) at 1300 pg/ml (normal range: 0-400 pg/ml). Chest radiograph showed increased markings with hilar congestion. Given the background of past vasculitis, raised inflammatory markers, and fever, a working diagnosis of active TA was reached. Since she had a new development of disproportionate dyspnea with raised dimer values, it was decided to proceed with a CT pulmonary angiogram (CTPA). She was immediately admitted to the medical intensive care unit and stabilized with oxygen therapy and other supportive measures. A cardiology opinion was sought and a 2D echocardiogram was done which did not show any evidence of pulmonary artery hypertension. Her CTPA showed large extensive multiple filling defect in bilateral pulmonary artery branches more marked on the right side, the findings compatible with acute bilateral pulmonary embolism (Figures 1, 2).

As part of the embolism work her bilateral lower limb venous Doppler did not show any evidence of deep vein thrombosis (DVT). Her CT images of the abdomen did not show any evidence of masses or lesions elsewhere which could have led to a thrombus formation. She was initiated on intravenous heparin with appropriate titration with coagulation markers as per the cardiologist's advice. In view of her respiratory failure, steroids were initiated at 1 mg/kg dose with a plan to titrate to steroid-sparing agents once she is out of danger. The decision not to thrombolyse was taken as there was no hemodynamic compromise or evidence of right ventricular strain. We suggested immunology opinion, angiography of major vessels, or positron emission tomography (PET) scan to look for the extent of active disease, but the patient deferred at the time due to social reasons. Gradually she got better and was weaned off oxygen support in two days. Meanwhile, her connective tissue markers (ANA, RA factor) and thrombophilia workup were done. Protein C, S deficiency, antithrombin 3, Factor 5 Leiden, and lupus anticoagulant all came to be normal. Her inflammatory markers and dimer values showed a decreasing trend. At the time of discharge, she could mobilize at a slow pace without any desaturation. She remains on oral anticoagulant drugs under close follow-up.

## Discussion

The disease was first reported by Japanese ophthalmology professor Mikito Takayasu in a patient who presented with vision loss [2]. Since then lots of cases have been reported worldwide. Epidemiologically it has been found more common in Asian countries [1].

TA is a chronic, nonspecific inflammatory disease of large and medium-sized vessels. It primarily involves the aorta and its branches. The clinical presentation is characterized by an acute phase with signs of inflammation and constitutional symptoms. Subsequently, after many months or years, a chronic phase can ensue with eventual fibrosis or occlusion of vessels [3]. Angiography is the gold standard for diagnosis. Now vascular imaging with contrast like CT, MRI, ultrasound, or PET imaging is a less invasive modality in assessing ongoing inflammation with clarity [4]. As per the American College of Rheumatology (ACR)/European League Against Rheumatism (EULAR) classification criteria, the absolute requirements for establishing the diagnosis of TA are age <60 years and evidence of vasculitis in the aorta and main branches by vascular imaging [5]. Additional criteria include female sex (+1), angina (+2), limb claudication (+2), arterial bruit (+2), reduced upper extremity pulse (+2), reduced pulse or tenderness of a carotid artery (+2), blood pressure difference between arms of ≥20 mm Hg (+1), number of affected arterial territories (+1 to +3), paired artery involvement (+1), and abdominal aorta plus renal or mesenteric involvement (+3). If the cumulative score is ≥5 points, the patient could be classified as having TA. The main complications of TA are due to ischemic events or vascular stenosis and occlusion [6].

TA involving the pulmonary arteries carries a prevalence ranging from 5% to 36% [7]. Pulmonary artery findings of TA can include stenosis, occlusion, aneurysm, infiltration, and pulmonary hypertension. Pulmonary artery involvement can also induce pulmonary parenchymal lesions such as mosaicing and infarction due to hypoperfusion, hemorrhage, bronchiectasis, cavitation, and pleural effusion. The presentation of pulmonary arteritis can be very nonspecific like a respiratory illness with repeated fever, chest pain, and hemoptysis with or without dyspnea. This often leads to misdiagnosis and further worsening to irreversible complications. Usually, pulmonary artery involvement may not correlate with disease activity. Pulmonary involvement can lead to pulmonary hypertension in 50% of cases, which bears a poor prognosis [8]. Usually, thrombus originates from the lower extremities or pelvis, reaches pulmonary vasculature, and causes pulmonary embolism. Pulmonary thromboembolism without evidence of DVT is called in situ pulmonary artery thrombosis (PAT). Pulmonary diseases, trauma, immunological, congenital, and hematological systemic diseases may lead to the formation of in situ PAT. The pathogenic factors causing in situ PAT are deemed as pulmonary vascular endothelial cell dysfunction, hypoxia, and inflammation. [9] Vessel wall inflammation, atherosclerosis, and hypercoagulability may be responsible for ischemic events in TA. The risk for cardiovascular and cerebrovascular events is less when compared to giant cell arteritis (GCA). There is a lack of consensus on giving lifelong aspirin to avoid critical events in GCA. EULAR and the British Society for Rheumatology (BSR) do not recommend routine use of antiplatelet or anticoagulant therapy unless in some indications like ischaemic heart disease, cerebrovascular disease, etc. In TA, aspirin may be recommended long-term if there is critical vessel stenosis or associated with cardiovascular comorbidities like hyperlipidemia or obesity.

Being an inflammatory disease, the main aim of treatment is to control inflammation and limit complications and progression. Corticosteroids are the mainstay of treatment. They are started usually at doses of 1-2 mg/kg to achieve remission and then tapered off. In case of relapse, other immunosuppressive agents such as methotrexate, azathioprine, or mycophenolate may be given. In patients who remain resistant and/or intolerant to these agents, biologic drugs including tumor necrosis factor inhibitors like rituximab and IL6 inhibitors like tocilizumab are alternatives [10]. In the presence of complications like critical arterial stenosis, endovascular procedures balloon angioplasty, or stent graft replacement being less invasive are preferred [11].

## Conclusions

Large vessel vasculitis if undiagnosed can pose life-threatening complications. In situ PAT in TA is a rare manifestation. Pulmonary artery involvement in the form of pulmonary hypertension and thrombosis usually presents with constitutional symptoms and need a high index of suspicion to reach a correct diagnosis. Identification at the right time can limit the inflammatory process and progression of the disease and the overall outcome of the patient.
